# Evaluation of Polymerization Efficacy in Composite Resins via FT-IR Spectroscopy and Vickers Microhardness Test

**DOI:** 10.15171/joddd.2015.041

**Published:** 2015-12-30

**Authors:** Tahereh-Sadat Jafarzadeh, Mohammad Erfan, Marjan Behroozibakhsh, Mostafa Fatemi, Reza Masaeli, Yashar Rezaei, Hossein Bagheri, Yasaman Erfan

**Affiliations:** ^1^Associate Professor, Department of Dental Biomaterials, Tehran University of Medical Sciences, Iranian Tissue Bank and Research Center, Tehran University of Medical Sciences Tehran, Iran; ^2^Associate Professor, Department of Pharmaceutics, School of Pharmacy, Shahid Bahashti University of Medical Sciences, Tehran, Iran; ^3^Assistant Professor, Research Center for Science and Technology in Medicine and Department of Dental Biomaterials, School of Dentistry, Tehran University of Medical Sciences, Tehran, Iran; ^4^Ph.D Candidate, Research Center for Science and Technology in Medicine and Department of Dental Biomaterials, School of Dentistry, Tehran University of Medical Sciences, Tehran, Iran; ^5^Assistant Professor, Department of Dental Biomaterials, Faculty of Dentistry, Tabriz University of Medical Sciences, Tabriz, Iran; ^6^Assistant Professor, Dental Materials Research Center, Faculty of Dentistry, Mashhad University of Medical Sciences, Mashhad, Iran; ^7^Dental Student, The International Branch of Shahid Behesti University of Medical Sciences and Health Services, Tehran, Iran

**Keywords:** Composite resins, Fourier transform infrared spectroscopy, hardness, polymerization

## Abstract

***Background and aims. ***Polymerization efficacy affects the properties and performance of composite resin restorations.The purpose of this study was to evaluate the effectiveness of polymerization of two micro-hybrid, two nano-hybrid and one nano-filled ormocer-based composite resins, cured by two different light-curing systems, using Fourier transformation infrared (FT-IR) spectroscopy and Vickers microhardness testing at two different depths (top surface, 2 mm).

***Materials and methods.*** For FT-IR spectrometry, five cylindrical specimens (5mm in diameter × 2 mm in length) were prepared from each composite resin using Teflon molds and polymerized for 20 seconds. Then, 70-μm wafers were sectioned at the top surface and at2mm from the top surface. The degree of conversion for each sample was calculated using FT-IR spectroscopy. For Vickers micro-hardness testing, three cylindrical specimens were prepared from each composite resin and polymerized for 20 seconds. The Vickers microhardness test (Shimadzu, Type M, Japan) was performed at the top and bottom (depth=2 mm) surfaces of each specimen. Three-way ANOVA with independent variables and Tukey tests were performed at 95% significance level.

***Results.*** No significant differences were detected in degree of conversion and microhardness between LED and QTH light-curing units except for the ormocer-based specimen, CeramX, which exhibited significantly higher DC by LED. All the composite resins showed a significantly higher degree of conversion at the surface. Microhardness was not significantly affected by depth, except for Herculite XRV Ultra and CeramX, which showed higher values at the surface.

***Conclusion.*** Composite resins containing nano-particles generally exhibited more variations in degree of conversion and microhardness.

## Introduction

Compositeresins opened new horizons for esthetics in restorative dentistry. In an adhesive procedure adequate photo-polymerization is extremely important for optimization of the physical, mechanical and clinical results of composite resins.^1^ Ideally, it is desirable for a dental composite resin to have all of its monomers polymerized during the polymerization reaction. However, this does not happen and a certain proportion of the reactive methacrylate groups remain unreacted as residual monomers.^2^

This is believed to be due to the loss of mobility and decreased reactivity of the polymer radicals after the polymer network began to form.^2^ Additionally, carbon-carbon double bonds occur even in diluted monomers, such as TEGDMA, which are supposed to have higher degree of conversion.^2,3^The filler content,^2^ size,^4^ shape,^4^ distribution and resin matrix^5^ affect the properties of composite resin materials. To achieve adequate polymerization of light-cured composite resins, sufficient light intensity and exposure time play an important role.^6^ It has been shown that light-curing duration, intensity and the type and mode of curing,^7,8^ as well as photoinitiator type of composite resin^9^ affect the degree of conversion, polymerization depth and microhardness of these restorations.

Different light-curing systems are used to initiate the polymerization reaction in composite resins, which contain photoinitiators such as camphorquinone (CQ). These systems include conventional quartz-tungsten-halogen (QTH) lamps and solid-state light-emitting diodes (LED). The broad absorption wavelength range of CQ (400-500 nm with a peak near 470 nm) matches that of filtered light emitted from QTH (400-500 nm). On the other hand, LED units are developed based on targeting the peak absorption wavelength of CQ, by emitting a relatively narrowband of light at 430-480 nm.^10^ This narrower band is considered advantageous because of the absence of QTH drawbacks, including excessive heating^9,10^ and declining power density over time due to bulb and filter aging. However, some authors stated that LEDs can produce as much heat as QTH lamps^8^ and the others have shown that the emitting lights at narrow wavelength spectra might fail to appropriately cure composite resin.^11^ Nevertheless, the efficacy of both units in polymerizing the composite resin seems to be sufficient.

Nanotechnology has led to the development of novel composite resin materials, nano-composites, which contain nano-filler particles. Also, a new type of organic-inorganic dental composite resin, based on the new organically modified ceramic, or ormocer, has been developed.^12^

To evaluate the efficacy of polymerization in composite resins, several types of laboratory tests are documented in the literature,^13^ which can be divided into direct and indirect methods. Indirect methods include microhardness measurement, optical microscopy and scraping testing. Some direct methods are differential thermal analysis (DTA), infrared spectroscopy and Raman spectroscopy. Fourier transform infra-red spectroscopy (FT-IR) technique evaluates the degree of conversion by comparing the vibration bands of the residual unpolymerized methacrylate C=C stretching mode at 1640 cm^-1^ to the aromatic C=C stretching mode at 1610 cm^-1^. FT-IR spectroscopy is based on the absorption of radiation in the infrared frequency range in accordance with the molecular vibrations of the functional groups contained in the polymer chain.^14^

Studies have shown that the degree of polymerization is lower in dental composite resins containing nano-fillers.^7,15^ On the other hand, there are inconsistent data about the influence of the light-curing unit type (QTH vs. LED) on the efficacy of polymerization.^16-18^ Due to increasing demand and marketing of nano-hybrid compositeresins, this study sought to evaluate and compare the effectiveness of polymerization of dental composite resins containing nano-scaled fillers, conventional micro-hybrid composite resins and a photo-polymerized ormocer using either QTH or LED light-curing units, by FT-IR and Vickers microhardness tests.

## Materials and Methods

The specifications of five commercially available light-cured composite resins used in this study are shown in Table 1. The specimens were photo-polymerized with LED (Demi, Kerr, USA) and QTH (Coltolux®75-Germany) light-curing units. The light output for both devices was tested by radiometers (LED, Demetron, 910724, Kerr, USA and Optilux, Model 100,10503, Kerr, USA, respectively), which registered over 1200 mW/cm^2^ for the LED Coltolux® and over 600 mW/cm^2^ for Coltolux® 75. Specimen preparation and method of cure conformed to manufacturer specifications.

**Table 1:** Specifications of materials used in this study

**Materials**	**Shade**	**Lot number**	**Composition**	**Manufacturer**
**CeramX Mono ** **(Ormocer)**	(M_2_)	806003117	Methacrylate modified polysiloxane (organically modified ceramic),dimethacrylate resin, ethyl-4(dimethylamino)benzoate, barium-aluminium-borosilicate glass(1.1-1.5 µm), methacrylate functionalized silicon dioxide nano filler(10 nm, mean nano filler size), Additives, stabilizers and catalysts, pigments	(DentsplyDeTrey), Konstanz, Germany
**Herculite Classic ** **(Microhybrid) **	(A2)	3423458	Bis-GMA, TEGDMA, Barium glass and silicon dioxide fillers, Additives, stabilizers and catalysts, pigments	Kerr Italia S.r.l.
**Tetric Ceram ** ** (Microhybrid)**	(A_2_)	J26729	Dimethacrylates, Barium glass filler, Ba-Al Fluorosilicate glass, Ytterbium trifluoride(0.7-1 ?m mean filler size), mixed oxide, highly dispersed silica, prepolymers, Additives, stabilizers and catalysts, pigments	(Ivoclar-Vivadent), Schaan, Liechtenstein
**TertricEvoceram ** **(Nanohybrid)**	(A_2_)	H32592	Dimethacrylates, Barium glass filler(550 nm mean particle size; range: 40 nm to 3000 nm), Ytterbium trifluoride, mixed oxide, prepolymers, Additives, stabilizers and catalysts, pigments	(Ivoclar-Vivadent), Schaan, Liechtenstein
**Herculite XRV Ultra (Nanohybrid)**	(A_2_)	3302434	Bis-GMA, TEGDMA, Prepolymerized filler, Silica nanofiller(20-50 nm nanoparticles), Barium submicron fillers(0.6 µm average size), Titanium Dioxide (TiO_2_) and pigments	Kerr Italia S.r.l.

### FT-IR Spectroscopy

Five cylindrical specimens (5 mm in diameter × 2 mm in length) were prepared from each composite resin, using a Teflon mold. Sample size was determined according to previous studies^19,20^ (α=0.05 and β=0.20).The composite surface was covered using a Mylar strip and the tip of the light-curing device was placed in contact with the top surface of the strip. Each specimen was polymerized for 20 seconds. Then a thin wafer with the thickness of 100 µm was sectioned at the top surface and 2mm from the top surface using a microtome (Buehler, Isomet, USA). The wafer specimens were then polished using #400 SiC paper to achieve a thickness of 70 µm. The cutting and grinding procedures were carried out under water cooling to prevent temperature rise in the specimens. The thickness was measured using a micrometer (Mitutoyo, Japan). A small amount of uncured specimen was placed between two polyethylene strips and pressed between two glass slides to obtain a thin film approximately 70 µm in thickness. The infrared spectrum of uncured sample and each wafer specimen were analyzed with an FT-IR spectrometer (Bruker Tensor 27, Germany) operating at 16 scans at 4cm^-1^ resolution. The range from 1000 to 2000 cm^-1^was scanned. Finally, the range from 1590 to 1660 cm^-1^was expanded. The spectra, recorded initially as the transmission mode, were converted to absorbance mode by the microprocessor of the spectrometer. The DC of each specimen was determined by comparison of the ratio of the aliphatic carbon–carbon double bond (C=C) with that of the aromatic component for the cured and uncured samples. The aliphatic C=C group has a characteristic IR absorption peak around 1636 cm^-1^. The aromatic C=C peak around 1608 cm^-1^ originates from the aromatic bonds of benzene rings in the monomer molecules and its intensity remains unchanged during the polymerization reaction. By using the change in the ratio of the aliphatic C=C to the aromatic C=C before and after curing, DC of composite resin was calculated by the following equation:

$$\left[DC=1-\frac{\;[abs\;(aliphatic\;C=C)/abs\;(aromatic\;C=C)]\;of\;polymer}{[abs\;(aliphaticC=C)/abs\;(aromatic\;C=C)]\;of\;monomer}\times 100\right]$$

### Vickers Microhardness Test (VMH)

Three cylindrical specimens^21^ (5mm in diameter × 2 mm in length) were prepared from each composite resin using the same above-mentioned Teflon mold. Each specimen was polymerized for 20 seconds. Vickers microhardness test (Shimadzu HMV; Shimadzu Corporation, Tokyo, Japan), was performed at the top (depth=0 mm) and bottom (depth=2 mm) surfaces of each specimen (three indentations for each specimen), using a 50-g load for 15 seconds.

### Statistical Analysis

A three-way ANOVA with independent variables, including composite resin brand (five variables), light sources (two variables), and depth from the surface (two variables), and Tukey test were performed at 95% significance level.

## Results

The mean values and standard deviations for degree of conversion and microhardness for the five composite resins, two light-curing systems, and two depths are showed in Tables 2 and 3. The DC values in Bis-GMA-based composite resins were calculated from 51.25% to 72.89%. Also, the DC values for CeramX were between 36.06% and 78.42%. The 3-way ANOVA for DC and VMH data showed that the factor of depth was significant with all the composite resins (P<0.001). The factor of light-curing system showed significant results only in CeramX mono specimens for both DC (P<0.001) and VMH (P<0.001), with the LED exhibiting significantly better results.

Regardless of light-curing system, micro-hybrid TetricCeram showed the highest bottom-to-top ratios for DC and VMH, whereas nano-hybrid CeramX mono showed the lowest bottom-to-top ratios for DC and VMH using QTH light-curing unit. Furthermore, the factor of depth significantly affected the DC (P<0.001) and VMH (P<0.001) in this composite resin. Nano-hybrid Herculite XRV exhibited the lowest bottom-to-top ratios for DC and VMH using LED light-curing unit and the factor of depth significantly affected the DC (P=0.04) and hardness (P=0.001) with this composite resin.

Comparison of the coefficient of variation (CV%) of mean values for degree of conversion showed greater variations in composite resins containing nano-scaled particles(Table 2). Among the micro-hybrid specimens, Herculite Classic showed more variations in DC. Regardless of the particle size and the light-curing mode, the results of this study indicated relatively greater variations in the DC for both Herculite Classic and Herculite XRV Ultra.

**Table 2:** Results for degree of conversion obtained from FT-IR analysis, at the top and bottom surface

		**Light sources**						P-Value^*^	
		**QTH**			**LED**				
**Composites**	**Depth(mm)**	**Mean (%) ** **(SD)**	**CV (%)**	**Bottom/Top ** **(%)**	**Mean (%) ** **(SD)**	**CV (%)**	**Bottom/Top ** **(%)**	Light Source	Depth
**CeramX Mono**	0	65.28 (2.60)	4	55.2	78.42 (2.53)	3.22	83	<0.001	<0.001
	2	36.06 (1.28)	3.54		65.1 (1.95)	3			
**Herculite Classic**	0	56.51(2.68)	4.74	81.2	58.31(2.14)	3.67	91.7	0.52	0.003
	2	45.88(5.63)	12.27		53.47(7.43)	13.89			
**Tetric Ceram**	0	72.89 (2.00)	2.74	98.3	72.41 (0.62)	0.85	96.5	0.082	0.008
	2	71.68 (1.60)	2.22		69.9 (0.78)	1.12			
**TetricEvoceram**	0	62.75 (3.34)	5.32	88.3	56.89 (7.00)	12.29	96.6	0.116	0.026
	2	55.38 (0.70)	1.27		54.93 (3.4)	6.2			
**Herculite XRV**	0	59.13(14.70)	24.86	91.4	67.64(2.70)	3.99	75.8	0.561	0.04
	2	54.04(13.02)	24.09		51.25(8.45)	16.48			
^* ^ANOVA - α= 0.05									

VMH values showed higher CV% for nano-hybrid TetricEvoCeram composite resin, especially with the use of an LED light-curing unit (Table 3). For the Herculite XRV, variations were relatively low with the use of the QTH light-curing unit. This composite resin showed up to more than three times greater variations at the bottom surface (depth of 2mm) in comparison with top surface. For the micro-hybrid Tetric Ceram and Herculite Classic, similar variations were seen for the hardness at the top and bottom surfaces.

**Table 3:** Results for microhardness testing, at the top and bottom surface

**Composite**		**Light Source**						**P-value**	
		**QTH**			**LED**				
	**Depth**	**Mean (SD) ** **(kg/mm^2^)**	**CV (%)**	**Bottom/Top (%)**	**Mean (SD)** **(kg/mm** ^ 2 ^ **)**	**CV (%)**	**Bottom/Top (%)**	Light Source	Depth
**CeramX**	0	56.60(6.05)	10.68	61.7	62.31(3.40)	5.44	88.5	0.001	0.001
	2	34.97(2.98)	8.52		55.12(5.59)	10.14			
**Herculite Classic**	0	57.52(2.47)	4.29	87.3	54.51(3.85)	7.04	78.3	0.059	0.004
	2	50.23(3.08)	6.13		42.69(6.19)	14.47			
**Tetric Ceram**	0	47.28(3.49)	7.38	89.6	43.18(3.12)	7.20	90.4	0.202	0.117
	2	42.36(7.80)	18.39		39.05(4.23)	10.83			
**Tetric** **EvoCeram**	0	50.96(1.89)	3.68	87.0	44.90(11.34)	25.25	87.0	0.266	0.225
	2	44.34(7.76)	17.48		39.07(8.73)	22.32			
**Herculite XRV**	0	41.71(0.98)	2.32	78.5	44.22(0.50)	1.10	69.9	0.869	<0.001
	2	32.75(3.20)	9.77		30.90(5.73)	18.51			

## Discussion

The results of the current study showed that the DC of micro-hybrid TetricCeram was significantly higher than other composite resins. These results are in accordance with those obtained by da Silva et al^7^ and Ribeiro et al,^15^ in which the nano-filled composite resin showed a lower DC. Furthermore, a relatively lower CV% was obtained with this composite resin. This result is also in agreement with da Silva et al,^22^ who reported that composite resins with nanoparticles showed a significantly lower light transmittance compared to micro-hybrid composites. Although the factor of depth had no significant effect on VMH, significantly higher DC was obtained at the top surface in TetricCeram. This may be due to much lower CV% of data resulting from FT-IR compared with VMH in this study, which makes small differences significant. The high bottom/top ratio and low CV% for both DC and VMH of TetricCeram composite resin may show the highly predictable rate of polymerization at the depth of two millimeters from the surface. Compared to Herculite XRV Ultra, lower CV% was obtained with Herculite Classic. Therefore, Herculite Classic may show more superior clinical results due to higher bottom/top ratios and lower coefficients of variation, a claim which can be further studied in the future, considering other clinical concerns such as postoperative pain and the risk for composite resin de-bonding as the DC increases.

Differences in the composition of materials and the light characteristics of light units,^3^ as well as the thickness of composite resins may result in significant variations in performance. The ratio of filler relative to the resin content is also important.^23^ Thus, the final DC of a resin may depend on the chemical structure of the dimethacrylate monomer and the polymerization conditions such as light intensity, photoinitiator type and concentration.^24^

In this study, the values calculated for the DC in composite resins containing bis-GMA-based matrices were between 51.25% and 72.89%, which is in agreement with the results of previous works for Bis-GMA resins orBis-GMA-based composite resins.^7,25^ CeramX, which is an ormocer-based composite resin, showed a wide spectrum of values for DC (36.06% to 78.42%), regardless of the depth and the light-curing system. Ormocers are organically modified ceramics, and form by functionalizing an alkoxysilane with a polymerizable group. Subsequently, hydrolysis and condensation reactions lead to an oligomeric Si-O-Si nanostructure which replaces the conventional monomers in composite resins.^26^

Using microhardness analysis, the efficacy of polymerization can be evaluated indirectly. Previous studies believe that microhardness testing is more sensitive in detecting small changes in monomer conversion compared to FTIR spectroscopy.^13^ Adequate in-depth polymerization needs a bottom-to-top VHN ratio to reach 80%, and this bottom-to-top VHN ratio corresponds to a 90% bottom-to-top DC ratio.^23^ Most of the composite resins reached that bottom-to-top threshold in this study. However, CeramX Monocured by QTH and Herculite XRV failed to produce appropriate bottom-to-top microhardness ratios, particularly using the LED unit. Different chemical nature of polymerizable matrix as well as the filler size and distribution may result in weaker bottom/top polymerization ratio in CeramX Mono. This investigation also demonstrated no significant differences in microhardness values using QTH or LED light-curing units, which is consistent with some previous studies.^16,27^

Consistent with the results of some other studies,^9,17^ this study showed no significant differences in DC and Vickers microhardness for both LED and QTH light-curing units, despite the different energy densities (24 and 12 J/cm^2^, respectively). However, the ormocer-based composite resin, CeramX Mono, had higher degree of conversion using the LED light-curing unit. As the absorption spectrum of each photoinitiator is specific, it has been mentioned that the influence of the type of the light-curing unit (QTH or LED) on the polymerization may depend on the photoinitiator type.^9^ Therefore, the higher energy output of LED units would not improve the polymerization rate, if the emitted light is not absorbed by the photoinitiator. All the composite resin materials used in the present study benefit from camphorquinone as a photoinitiator. Therefore, any differences in the polymerization following curing by QTH or LED must be discussed in terms of the effect of other variables on the photo-activation of polymerization. This study showed that, using LED or QTH, the polymerization efficacy of composite resins was not significantly different in terms of DC and VMH, except for CeramX Mono, which showed higher DC and VMH using LED photo-curing .The different behavior of CeramX Mono might be attributed to modifications in the resin matrix. It seems that a greater temperature rise during photo-polymerization using QTH vs. LED light-curing units^18^ may compensate for lower energy output since it has been shown that raised temperature can affect the polymerization behavior of dimethacrylate-based materials. However, high-power LED unit has certain advantages over the halogen curing unit because it is cordless, smaller and lighter, with a whisper-quiet fan.

Generally the VMH values were coherent with DC in this study, which is consistent with previous studies.^28,29^ However, since each technique is sensitive to different variables, they cannot always be used interchangeably and the hardness number cannot predict the degree of conversion when comparing different resins.^28^

Considering the results of this study, it may be concluded that the nano size of particles may be responsible for scattering of the light. Also, nano particles are more effective in reducing DC in deeper layers. However, it does not mean that nano-hybrid composite resins do not have efficient performance as micro-hybrid formulations. Precise technique, adequate light emission (i.e. minimum distance of light-curing unit tip, periodic checking of light intensity of device and adequate time of illumination), in combination with appropriate incremental technique for composite placement, might play a more determined role in obtaining successful results.

## Conclusion

Considering the limitations of this in vitro study, it can be concluded that, although both nano-hybrid and micro-hybrid composite resins showed an appropriate rate of polymerization, more variations may be obtained in the polymerization of nano-hybrid composite resins.

## Acknowledgments

This study was supported by a grant from the Research Center for Science and Technology in Medicine (RCSTIM) of Tehran university of Medical Sciences.
